# Hyperbaric oxygen preconditioning for prevention of acute high-altitude diseases: Fact or fiction?

**DOI:** 10.3389/fphys.2023.1019103

**Published:** 2023-01-25

**Authors:** Jiuhong You, Xinxin Chen, Mei Zhou, Hui Ma, Qiaoling Liu, Cheng Huang

**Affiliations:** ^1^ Rehabilitation Medicine Center, West China Hospital, Sichuan University, Chengdu, China; ^2^ Key Laboratory of Rehabilitation Medicine in Sichuan Province, West China Hospital, Sichuan University, Chengdu, China; ^3^ School of Rehabilitation Sciences, West China School of Medicine, Sichuan University, Chengdu, China; ^4^ Institute of Cardiovascular & Medical Sciences, University of Glasgow, Glasgow, United Kingdom

**Keywords:** hyperbaric oxygenation, acute mountain sickness, high-altitude cerebral edema, preconditioning, high-altitude pulmonary edema

## Abstract

Acute high-altitude diseases, including acute mountain sickness (AMS), high-altitude cerebral edema (HACE), and high-altitude pulmonary edema (HAPE), have been recognized as potentially lethal diseases for altitude climbers. Various preconditioning stimuli, including hyperbaric oxygen (HBO), have been proposed to prevent acute high-altitude diseases. Herein, we reviewed whether and how HBO preconditioning could affect high-altitude diseases and summarized the results of current trials. Evidence suggests that HBO preconditioning may be a safe and effective preventive method for acute high-altitude diseases. The proposed mechanisms of HBO preconditioning in preventing high-altitude diseases may involve: 1) protection of the blood-brain barrier and prevention of brain edema, 2) inhibition of the inflammatory responses, 3) induction of the hypoxia-inducible factor and its target genes, and 4) increase in antioxidant activity. However, the optimal protocol of HBO preconditioning needs further exploration. Translating the beneficial effects of HBO preconditioning into current practice requires the “conditioning strategies” approach. More large-scale and high-quality randomized controlled studies are needed in the future.

## 1 Introduction

With the rapid development in transportation from low to high altitudes and the growing interest in adventure travel, more than 100 million people now travel to high altitudes for work or pleasure annually. The decreased barometric pressure and ambient oxygen tension at high altitudes can trigger a range of physiological responses, leading to maladaptive reactions and acute altitude illness (Luks, 2015), including acute mountain sickness (AMS), high-altitude cerebral edema (HACE), and high-altitude pulmonary edema (HAPE) (Clark and Sheraton, 2021). AMS is by far the most common high-altitude disease, with a reported incidence of more than 25% and 50% in people ascending to 3,500 and above 6,000 m, respectively (Meier et al., 2017). HACE rarely occurs at altitudes lower than 4,000 m, with an incidence of 0.5%–1% between 4,200 and 5,500 m. The incidence of HAPE is less than 0.2% if individuals spend three days climbing to an altitude of 4,000–5,000 m, but can be up to 7% in a single-day climb (Luks et al., 2017). Moreover, 85%–100% of individuals diagnosed with HACE also develop HAPE (Turner et al., 2021).

Hyperbaric oxygen therapy (HBOT) refers to the inhalation of 100% oxygen at a pressure higher than normal atmospheric pressure (Jain, 2016). A growing body of studies demonstrated that HBO preconditioning might prevent high-altitude diseases. Therefore, this review aimed to address whether and how hyperbaric oxygen (HBO) preconditioning may affect acute high-altitude diseases, and to summarize the results of current trials.

## 2 High-altitude diseases

### 2.1 Clinical manifestation and diagnosis

According to the 2018 Lake Louise AMS Score (Roach et al., 2018), AMS was defined as the presence of headache in an unacclimatized person who had recently arrived at an altitude above 2,500 m, along with the presence of one or more of the following: gastrointestinal symptoms (anorexia, nausea, or vomiting), fatigue or weakness, dizziness or light-headedness. HACE, which usually occurs between 24 and 72 h at altitudes above 4,000 m, is characterized by neurological findings such as ataxia, decreased consciousness, and slurred speech (Jensen and Vincent, 2022). The classic evaluation of HACE includes an abnormal neurological examination, in which ataxia is usually the earliest finding. Laboratory tests may reveal an increased white blood cell count and lumbar puncture may show open pressure elevation. Magnetic resonance imaging may demonstrate a distinct swelling and hypertonicity of the posterior spleen and of the corpus callosum on T2 images (Hackett and Roach, 2004).

HAPE may occur within one to five days after reaching an altitude above 2,500 to 3,000 m, with the symptoms of excessive exertional dyspnea, cough, chest tightness, and decreased exercise performance (Luks et al., 2017). It would also have any two of the following findings: tachycardia, central cyanosis, rales/hiss, and shortness of breath. If detectable, a chest x-ray (CXR) may demonstrate patchy alveolar infiltration with normal mediastinal/cardiac size, and an ultrasound may demonstrate B-lines indicating pulmonary edema. Electrocardiogram might show right axis deviation and/or signs of ischemia. Rapid correction of clinical status and percutaneous arterial oxygen saturation with supplemental oxygen is a pathognomonic feature of HAPE in patients with infiltrates on CXR films. Even with laboratory tests, their role is limited and clinicians should always take into account the coexistence of AMS and/or HACE (Jensen and Vincent, 2022).

### 2.2 Pathophysiological processes

The hypobaric hypoxic environment at high altitudes has a great impact on human physiological functions and physical activities (Agrawal et al., 2017). Hyperventilation, polycythemia, hypoxic pulmonary vasoconstriction, alterations in the oxygen affinity of hemoglobin, a rise in oxidative enzymes, and an increased concentration of capillaries in peripheral muscle are classical responses of the body to high altitude. For AMS patients, arterial oxygen saturation (SaO_2_) and tension (PaO_2_) are a bit lower and the alveolar-arterial oxygen tension is a bit greater at high altitudes compared to healthy controls (Luks et al., 2017).

The pathogenesis of AMS and HACE remains controversial, but some evidence suggests that it is induced by elevated intracranial pressure (Dipasquale et al., 2016). Some experts believe that increased cerebral blood flow raises central nervous system pressure. Low arterial PaO_2_ leads to cerebral vasodilation, despite the opposite effect of reduced arterial partial pressure of carbon dioxide that accompanies hyperventilation. Other causes such as increased microvascular permeability and abnormal sodium and water balance may also cause AMS (West, 2012). Matrix metalloproteinase-9 (MMP-9) causes blood-brain barrier (BBB) destruction, brain edema, and neuroinflammation (Pan et al., 2020).

HAPE occurs as a response of the vasculature of the lungs to hypoxia. At high altitude, the body reacts to hypoxia by hyperventilating, which is known as the hypoxic ventilatory response. In hypoxic conditions, the reduced supply of nitric oxide (NO) and prostaglandin E2 (Bian et al., 2022) can cause excessive rise in pulmonary artery pressure (PAP). Increased PAP prior to the development of edema is the key pathophysiological factor of HAPE (Mulchrone et al., 2020).

## 3 Measures to improve high-altitude endurance

A slow ascent is the primary strategy for preventing altitude diseases (Luks et al., 2017). Furthermore, pharmaceutical agents (Ritchie et al., 2012) such as acetazolamide, calcium-channel blockers, and phosphodiesterase 5 inhibitors contribute to the prevention and treatment of acute high-altitude diseases. The Wilderness Medical Society has published guidelines on the use of hyperbaric chambers for treating severe AMS and HACE (Luks et al., 2019), but symptoms can recur when the patient leaves the chamber on the highlands. Therefore, the question remains whether it would be better to use HBO preconditioning to improve individual high-altitude endurance and prevent AMS.

## 4 HBO preconditioning for high-altitude diseases

Numerous studies have explored the effectiveness of HBO preconditioning for high-altitude diseases (study characteristics are shown in Table 1).

**TABLE 1:** Studies on hyperbaric oxygen preconditioning in preventing acute high-altitude diseases.

Author	Study subject	Intervention (N)	Control (N)		HBO regimen				Outcome	Result
					Pressure	Duration	Frequency	Number		
Cui et al. (2007c)	Animal	41	28		2.0 ATA	60 min	Once per day	2 to 5	NO, NOS	HBO-PC increased NO levels, improved blood circulation and reduced damage to hypoxic tissues.
Bu et al. (2021)	Human	29	31		0.11 MPa	90 min	Once per day	1	attention network test	HBO-PC improved orientation attention, but not alert or conflict attention.
Feng et al. (2017)	Human	6			2.0 ATA	120 min	Once per day	7	Tei index, LVEF, cTnI	HBO-PC can abate postexercise myocardial damage and enhance cardiac function.
Xiao and Hu. (2017)	Human	10	10		0.22 MPa	120 min	Once per day	5	LLSS	HBO-PC could improve the oxygen tolerance of the human body at high altitudes and have a preventive effect on AMS.
Cui et al. (2009)	Human	10			0.2 MPa	60 min	Once per day	1	pH, PaCO_2_, AaDO_2_, SaO_2_	HBO-PC can improve gas exchange, enhance oxygenate function, and accelerate the clearance of stacked lactic acid in the human body at high altitudes.
Wang et al. (2008)	Human	20	20		unknown	4 h	Once per day	3	GJB 1098-91, vital signs SaO_2_, Hb	HBO-PC could improve physiological function, inhibit the generation of free radicals of the body in hypoxic conditions, and reduce the incidence of AMS.
Hu et al. (2006)	Animal	14	14	14	0.25 MPa	60 min	Once per day	5	rCBF, PbtiO_2_	HBO-PC is neuroprotective against craniocerebral injury at high altitude, it increases rCBF and PbtiO2, reduces brain edema and improves neurological function.
Liu et al. (2007)	Animal	24	26	8	2.0 ATA	75 min	Once per day	3	IL-6, MMP-9	HBO-PC had no significant effect on the intra-arterial IL-6 and MMP-9 levels in rats exposed to acute hypobaric hypoxic conditions compared to the hyperbaric and normobaric preconditioning groups.
Cui et al. (2007b)	Human	41	48		2.0 ATA	60 min	Once per day	2-5	SOD, MDA, BLA, BUN	HBO-PC improved antioxidant activity and reduced blood lactate in altitude climbers.
Cui et al. (2008)	Human	21	28		2.0 ATA	60 min	Once per day	2-5	SOD, MDA, BLA, BUN, NO, NOS	HBO-PC could enhance the activity of anti-oxidase and the clearance of lactic acid, and anti-fatigue effect could last for eight days.
Liu et al. (2011)	Animal	16	16	8	2.0 ATA	75 min	Once per day	3	IL-6, MMP-9	HBO-PC had no preventive effect against AMS.
Ma G. et al. (2008)	Human	21	18		2.2 ATA	60 min	Once per day	2	PR, SaO_2,_ GJB 1098-91	In the control group, the AMS score and PR increased, and SaO_2_ decreased compared to the HBO-PC group; HBO-PC had a preventive effect on AMS.
Liang et al. (2007)	Animal	16	16	16	2.0 ATA	75 min	Once per day	3	IL-6, NE	HBO-PC could improve oxygen dispersion, enhance aerobic metabolism, produce more ATP, improve hypoxic tolerance and alleviate injury in the body.
Ma. (2007)	Human	20	20		2.0 ATA	70 min	Once per day	2	memory test, hypoxia challenge test and retinal circulation changes, dark adaptation, SaO_2_	Patients with HBO-PC had a better performance on the memory test and dark adaptation than untreated patients, which can retain for about one week. HBO-PC could prevent AMS.
Ma Y et al. (2008)	Human	30	30		2.0 ATA	70 min	Once per day	2	dark adaptation, SaO_2_, health state of retina	HBO could effectively improve brain-body physiological functions of individuals at altitude of 3700 m and over 5000 m, and effectively relieve hypoxic status.
Li et al. (2015)	Human	8	-		2.0 ATA	80 min	Once per day	7	DA, E, NE, ACTH	HBO-PC can elevate the plasma expression of DA, E, NE, and ACTH, and then speed up the establishment of a new balance of homeostasis to adapt to the acute hypoxia at high altitude.
Li et al. (2011a)	Animal	24	33	33	2.0 ATA	60 min	Once per day	5	AQP1, AQP5,	HBO-PC could decrease AQP1 and AQP5 levels in the lung, thus prevent high-altitude pulmonary injury.
Gao et al. (2008)	Human	20	29	20	2.0 ATA	60 min	A session per day	5	SOD, MBA	HBO-PC could increase SOD and decrease MDA, thus prevent AMS.
Cui et al. (2008)	Animal	6	7		0.25 MPa	60 min	A session per day	2	HR, PAP, RVSP, RVEDP, ±dp/dt_max_	HBO-PC decreased the HR, RVSP, PAP significantly, and increased dp/dt_max_ in rats, thus improve the haemodynamics and protect cardiopulmonary function in acute hypoxia rats.
Pan et al. (2008)	Animal	44	10	8	0.2 MPa	60 min	A session per day	5	CGRP, ET	HBO-PC did not affect CGRP, but increased ET, which may promote the body’s high-altitude acclimatization.
Wang et al. (2009)	Human	10	10		0.2 MPa	60 min	A session per day	2	Cognitive and physical test, SaO_2_, PR	HBO-PC group had improved cognitive and physical performance, higher SaO_2_ and lower PR.
Li et al. (2010)	Animal	15	15	8	0.2 MPa	60 min	A session per day	5	AQP-1, wet-to-dry weight ratio and morphology of the lung	The lung injury scores of the HBO-PC group were much lower than those of the HAPE group, and that AQP-1 and AQP-1 mRNA for the HBO group were significantly higher than those of the HAPE group.
Li et al. (2011b)	Animal	15	15	8	0.2 MPa	60 min	A session per day	5	AQP-5, wet-to-dry weight ratio and morphology of the lung	The lung injury scores of the HBO-PC group were much lower than those of the HAPE group, and that AQP-5 and AQP-5 mRNA for the HBO group were significantly higher than those of the HAPE group.
Ma et al. (2008)	Human	14	14	14	0.2 MPa	70 min	A session per day	2	HR, SaO_2_, GJB 1098-91	HBO-PC reduced HR and increased SaO_2_, and alleviated the AMS symptoms.
Wang et al. (2012)	Animal	12	12	8	0.2 MPa	45 min	A session per day	5	AQP-1, AQP-5, morphology of the lung	HBO-PC could upregulate AQP-1 and AQP-5, and prevent HAPE.
Cui et al. (2014)	Animal	30	20		0.2 MPa	60 min	A session per day	7	Water contents, morphology of the lung and heart	The water contents were decreased markedly in HBO-PC group, and the morphology of lungs and heart were more intact in HBO-PC group.
Xiao and Hu. (2017)	Human	10	19		0.22 MPa	60 min	A session per day	5	AMS incidence	The incidence of AMS of HBO preconditioning group and control group was 30% and 90%.

Abbreviations: AMS, acute mountain sickness; HBO, hyperbaric oxygen therapy; HBO-PC, hyperbaric oxygen preconditioning; NO, nitric oxide; NOS, nitric oxide synthase; LVEF, left ventricular ejection fraction; CK-MB, serum creatine kinase isoenzymes-MB; cTnI, cardiac troponin I; LLSS, the lake louise acute mountain sickness scoring system**;** PaO_2_, partial pressure of oxygen; PaCO_2_, partial pressure of carbon dioxide; AaDO_2_, alveoli - arterial oxygen differential pressure; SaO_2_, oxygen saturation; Hb, hemoglobin; rCBF, regional cerebral blood flow; PbtiO_2_, brain tissue oxygenation; IL-6, interleukin 6; MMP-9, matrix metalloproteinase-9; SOD, superoxide dismutase; MDA, malondialdehyde; BLA, blood lactic acid; BUN, blood urea nitrogen; NE, neutrophilic granulocyte; GJB, 1098-91, the principle of diagnosis and treatment of acute mountain sickness in China; DA, dopamine; E, epinephrine; NE, norepinephrine; ACTH, adrenocorticotropic hormone; AQP1, aquaporin 1; AQP5, aquaporin 5; CGRP, calcitonin gene related protein; ET, endothelin; PR, pulse rate; HR, heart rate.

### 4.1 HBO preconditioning for human responses to high-altitude and AMS

HBO preconditioning may alleviate the body’s hypoxic response at high altitudes, improve cognitive and physical function, and prevent AMS.

First, HBO preconditioning could improve the SaO_2_ (Wang et al., 2009; Yang et al., 2014) and PaO_2_ level, reduce heart rate and pulse rates, alleviate AMS symptoms in humans, and the effects could last for a week (Ma G. Q. W. et al., 2008). Moreover, under hyperbaric conditions, one can dissolve enough oxygen, that is 6 vol% in the plasma, to satisfy the typical physical needs (Jain, 2016). HBO preconditioning increases plasma expression of dopamine, epinephrine, and adrenocorticotropic hormone, and then accelerates the establishment of a new homeostasis that promotes the acclimatization of the organism in a high-altitude environment (Li et al., 2015). Pan et al. (2008) compared the preconditioning effects between hypoxia preconditioning and HBO preconditioning, and observed that both methods increased endothelin and promoted altitude acclimatization.

Second, HBO preconditioning could improve individuals’ cognitive and physical performance at high altitudes. Studies (Ma, 2007; Wang et al., 2009) found that people receiving HBO preconditioning twice had better memory and responsiveness ability compared to control group. HBO preconditioning could also reduce lactic acid levels in the body, help in reducing fatigue and refresh the workers. Shi et al. (Shi Lu et al., 2015) observed that three to seven sessions of HBO preconditioning increased glucose levels after physical loading and reduced blood lactate (BLA), lactate dehydrogenase and Na + -K + -ATPase levels at the same time. Moreover, three-day HBO preconditioning showed the best efficacy. It turns out that short-term HBO preconditioning can significantly improve energy metabolism in individuals under physical load at high altitude, accelerate glycogenolysis, and lead to better utilization of oxygen by muscles, thus alleviating fatigue and enhancing work efficiency.

Regarding body organs, the photoreceptor system of the eye is the most sensitive to hypoxia. Previous studies showed that HBO preconditioning could improve the health of the retina, dark adaptation, and orienting attention (Ma G. Q. W. et al., 2008; Shi Lu et al., 2015). There is a functional decline in coordinated movement when cerebral hypoxia occurs, and HBO preconditioning can enhance body coordination. A study of Liang et al. (2007) showed that HBO preconditioning can improve the body’s blood oxygen dispersion, enhance aerobic metabolism, and generate more ATP. Therefore, it can enhance the body’s ability to tolerate hypoxia and reduce body damage. Two HBO pretreatments reversed tissue hypoxia and improved post-exercise haemodynamic indexes (Cui et al., 2007a), and this effect lasted for approximately a week. Xiao and Hu. (2017) found that the incidence of AMS was lower after five HBO pre-treatments compared to the control group (30% vs. 90%). Liu et al. (2011) found that three HBO pretreatments reduced the levels of interleukin 6 (IL-6) and MMP-9 in mice with AMS, but the difference was not significant. Therefore, further studies are needed to confirm the prophylactic effect of HBO preconditioning.

### 4.2 HBO preconditioning for HACE and HAPE

Studies on the prevention of HACE and HAPE with hyperbaric oxygen preconditioning are mainly animal studies.

Previous studies found that heat shock protein (HSP) 70-mediated HBO preconditioning could prevent high-altitude brain edema, cognitive deficits, and hippocampal oxidative stress in rats (Lin et al., 2012a; Lin et al., 2012b). Zhou et al. (2017) found that HBO preconditioning significantly alleviated the increase of BBB permeability and water content in rats caused by high-altitude hypoxia, and had a preventive effect on HACE.

As for the preventive effects of HBO preconditioning on HAPE, the mechanisms may involve HSP70 and aquaporin (AQP) (Tsai et al., 2014; Lin et al., 2015). AQP1 and AQP5 provide the main pathway for water transport through the pulmonary microvascular endothelium and are involved in the pathogenesis of pulmonary edema. Studies showed that HBO preconditioning alleviated the upregulation of AQP1 and AQP5 in the lung, thus preventing HAPE (Li et al., 2010; Li et al., 2011a; Li et al., 2011b). Cui et al. (2008) reported that HBO preconditioning significantly increased heart rate, PAP, right ventricular systolic pressure, right ventricular end-diastolic pressure, and the maximum rate of rise/decline of right ventricular pressure in rats at high altitude. In another study by Cui et al. (2014), water contents in the tissues of the rats in the HBO preconditioning groups were all decreased markedly than those in the control groups. In addition, the morphology of lungs and heart were more intact in HBO preconditioning group. These studies may suggest that HBO preconditioning can improve hemodynamics and protect cardiopulmonary function in acute hypoxic rats.

### 4.3 The proposed mechanisms of HBO preconditioning in preventing high-altitude diseases

**FIGURE 1:**
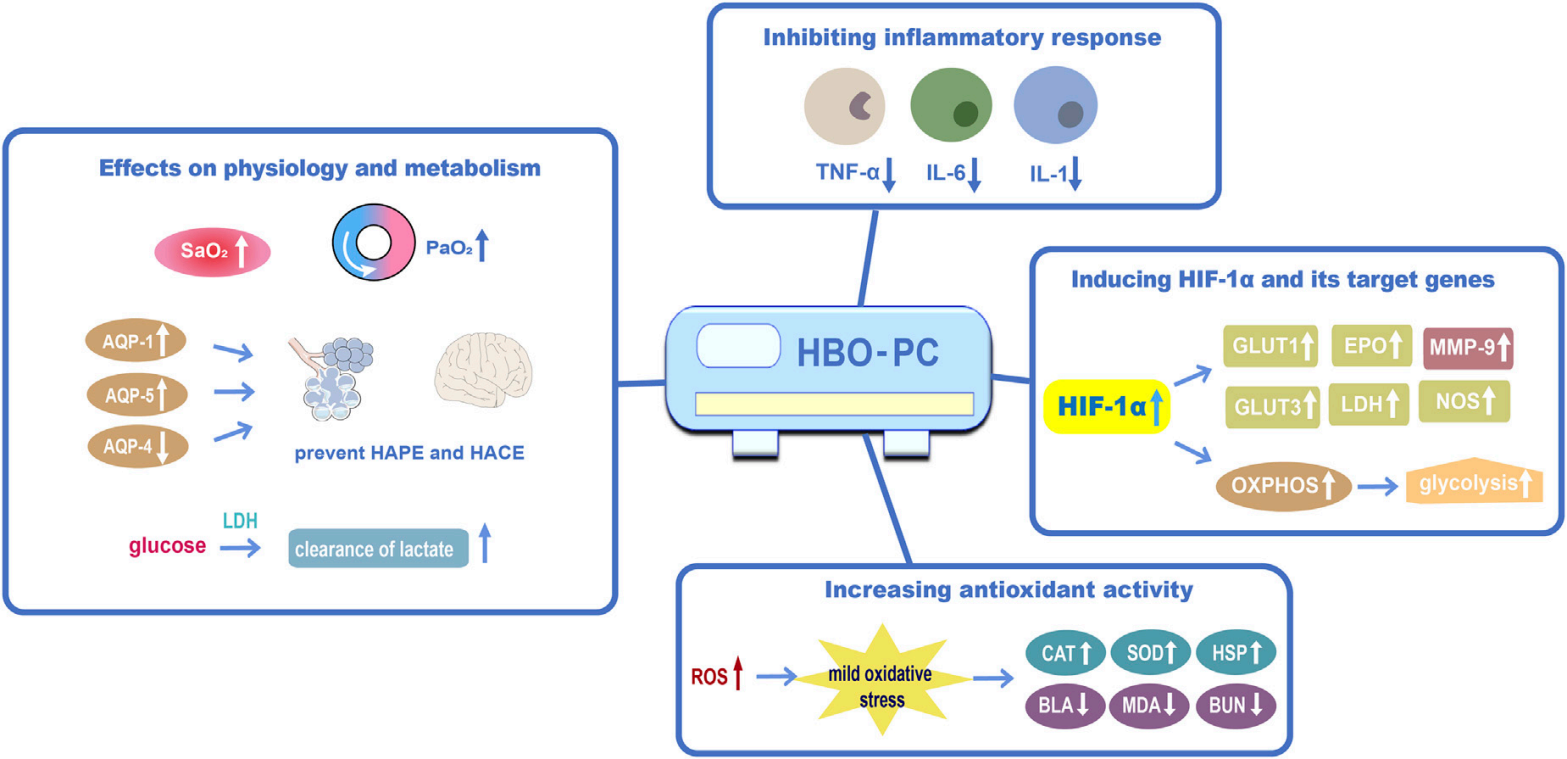
The proposed mechanisms of HBO preconditioning in preventing high-altitude disease (1) Effects of HBO preconditioning on physiology and metabolism against acute high-altitude diseases; (2) HBO preconditioning may inhibit inflammatory responsiveness by reducing apoptosis and shifting mitochondria; (3) HBO preconditioning may induce HIF-1α and its target genes; (4) HBO preconditioning could increase antioxidant activity. Abbreviations: HBO-PC, hyperbaric oxygen preconditioning; PaO_2_, partial pressure of oxygen; SaO_2_, oxygen saturation; AQP-1, aquaporin 1; AQP-5, aquaporin 5; AQP-4, aquaporin 4; IL-6, interleukin 6; HIF-1α, hypoxia-inducible factor-1α; GLUT1, glucose transporter 1; EPO, erythropoietin; MMP-9, matrix metalloproteinase-9; GLUT3, glucose transporter 3; NOS, Nitric oxide synthase; OXPHOS, oxidative phosphorylation; ROS, reactive oxygen species; CAT, catalase; HSP, heat-shock protein; BLA, blood lactic acid; MDA, malondialdehyde; BUN, blood urea nitrogen.

Based on the existing evidence, we propose some potential mechanisms for HBO preconditioning in the prevention of high-altitude disease (Figure 1).

#### 4.3.1 Inhibiting inflammatory responses

HBO preconditioning may play an important role in inhibiting inflammatory responses to prevent high-altitude diseases. Qi et al. (2013) reported that HBO preconditioning reduced inflammatory factors such as interleukin 1, tumor necrosis factor-α, and IL-6 in rats. HBO provides oxygen and thus promotes NO synthesis. Cui et al. (2007c) found that HBO preconditioning could boost blood circulation, increase NO concentrations, and reduce hypoxic tissue injury in the human body at high altitudes, and this effect can last for more than eight days.

#### 4.3.2 Generating HIF-1α

HIF-1 is a crucial modulator for inducing genes that promote cellular adaptation and survival under hypoxic conditions (Semenza, 1998). Some pharmacotherapies have been proven to prevent AMS by regulating the HIF-1 signaling pathway (Wang et al., 2019; Yan et al., 2021). Recent studies have shown that HBO can increase the expression of HIF-1α by stabilizing and activating it (Salhanick et al., 2006; Duan et al., 2015; Salmón-González et al., 2021).

Theoretically, hyperoxia should reduce HIF, then this raises a question regarding how HBO increases HIF. The specific mechanisms remain unclear, but the following hypotheses have been discussed. First, the brain tissue undergoes relative hypoxia following HBO preconditioning, as the oxygen level is reduced to 21% of normal levels after the 100% O_2_ level in the HBO chamber. Hence, repeated HBO preconditioning may generate a hyperoxia and hypoxia cycle and induce an accumulation of HIF-1α (Fratantonio et al., 2021). Second, in addition to hypoxia, other stimuli such as cytokines, growth factors, hormones, and viral proteins can induce HIF-1 (Gu et al., 2008). Third, a possible mechanism is the production of ROS by HBO preconditioning. The stabilization of HIF-1α is related to increased ROS (Guzy et al., 2005). Intermittent hyperoxia produces a state that mimics hypoxia through a reduced ROS/clearance capacity ratio (Hadanny and Efrati, 2020). These findings suggest that HBO preconditioning may increase HIF in a delayed manner to promote altitude acclimation.

#### 4.3.3 Increasing antioxidant activity

Acute hypobaric hypoxia increased the malondialdehyde (MDA) level (Agrawal et al., 2017). Previous studies found that HBO preconditioning could reduce MDA, blood urea nitrogen levels, and BLA in high altitudes. In addition, HBO preconditioning could increase superoxide dismutase, thus we can infer that HBO preconditioning could increase the activity of antioxidants and degrade serum lactate levels, and has a preventive effect on AMS (Cui et al., 2007b; Gao et al., 2008).

### 4.4 The protocol of preconditioning for preventing acute high-altitude diseases

The common HBO preconditioning pressure in these included studies was two ATA. The number of HBO preconditioning sessions ranged from one to seven, while two to five sessions were the common choices. Almost every study chose to do the HBO preconditioning once a day. The neuroprotective effect induced by HBO preconditioning is short-term and usually disappears in approximately two weeks (Qi et al., 2020). Therefore, climbers should preferably be exposed to HBO preconditioning within two weeks before climbing. The optimal regimen of HBO preconditioning for preventing high-altitude diseases still needs further exploration.

### 4.5 Safety of HBO preconditioning

HBOT is used in an increasing number of conditions and is seen to be a generally safe treatment. The possible side effects of HBO involve barotrauma, oxygen poisoning, visual abnormalities, and claustrophobia (Jain, 2016). Middle ear barotrauma, which has been reported in up to 2% of treated individuals, is by far the most common and benign side effect. It can be avoided or decreased by teaching autoinflation skills or by inserting tympanostomy tubes. Claustrophobia is another common symptom that calls for assurance, counseling, and even medication. Progressive myopia is typically temporary and reversible after stopping HBO sessions. Pulmonary dyspnea with cough and inspiratory pain are caused by oxygen toxicity, from the multiple exposures required for chronic treatments. Rarely, at greater oxygen pressures, during acute therapies in acidotic patients do more severe seizures occur. A retrospective analysis found that rigorous operational procedures (chamber monitoring and assessment prior to HBO treatment) were essential to increase the safety of patients. When used properly, HBOT could be one of the safest medical techniques (Hadanny et al., 2016).

## 5 Discussion

To translate HBO preconditioning paradigm to the clinic, it is critical to identify vulnerable groups at high risk for acute high‐altitude diseases. Previous studies have shown that AMS history, level of residence, or recent high-altitude exposure and ascending rate are significant predictors of high-altitude diseases (Erik and Swenson, 2014). Obesity (Yang et al., 2015), age, and gender may be associated with the incidence of high-altitude disease (Maloney and Broeckel, 2005). However, exact risk factors are yet to be identified.

For climbers, it is convenient to take HBO preconditioning. Hyperbaric chambers can be categorized into monoplace chambers and multiplace chambers. The monoplace chamber can only accommodate one person, and the multiplace chamber allows for the treatment of several patients at the same time (Lind, 2015). There are also portable chambers (Butler et al., 2011). Those portable chambers are for single use and can fit into a backpack when deflated, which are carried on most high-altitude expeditions. The drawback is that they are very limited in the pressure, and the highest common pressure that can be achieved is about 1.4 ATA. The portable chamber is mainly used in the treatment of high-altitude diseases. As it is an U.S. Food and Drug Administration-approved indication, HBOT can be implemented quickly once the treatment has been optimized for humans (Lippert and Borlongan, 2019).

To date, HBOT has been widely used in the treatment of high-altitude disease; however, the preventive role of HBOT pretreatment has been largely overlooked. To the best of our knowledge, this is the first study to review the effect of HBO preconditioning in preventing acute high-altitude diseases. Both animal studies and clinical studies showed that HBO preconditioning may be an effective modality to prevent high-altitude diseases. The proposed mechanism of HBO preconditioning in preventing high-altitude diseases may involve: 1) the protection of the BBB and prevention of brain edema, 2) the inhibition of inflammatory responses, 3) the induction of HIF-1α and its target genes, and 4) the increase in antioxidant activity. Based on the current results, multi-sessions of hyperbaric oxygen preconditioning may have a better preventive effect on acute altitude disease compared to a single session. The combined effect of pre- and post-climbing HBO use also warrants further study. With further confirmed role and the development of an optimal protocol of hyperbaric oxygen preconditioning, it will be beneficial to lower the morbidity of altitude diseases, and reduce the life, health and economic loss.

## Data Availability

The original contributions presented in the study are included in the article/supplementary material, further inquiries can be directed to the corresponding author.
